# An Interactive Real-Time Locating System Based on Bluetooth Low-Energy Beacon Network †

**DOI:** 10.3390/s18051637

**Published:** 2018-05-21

**Authors:** You-Wei Lin, Chi-Yi Lin

**Affiliations:** Department of Computer Science and Information Engineering, Tamkang University, New Taipei City 25137, Taiwan; 604420066@s04.tku.edu.tw

**Keywords:** Bluetooth low energy, location-based service, mesh network, internet of things

## Abstract

The ubiquity of Bluetooth-enabled smartphones and peripherals has brought tremendous convenience to our daily life. In recent years, Bluetooth beacons have also been gaining popularity in implementing a variety of innovative location-based services such as self-guided systems in exhibition centers. However, the broadcast-based beacon technology can only provide unidirectional communication. In case smartphone users would like to respond to the beacon messages, they have to rely on their own mobile Internet connections to send the information back to the backend system. Nevertheless, mobile Internet services may not be always available or too costly. In this work, we develop a real-time locating system based only on the Bluetooth low energy (BLE) technology to support interactive communications by combining the broadcast and mesh topology options to extend the applicability of beacon solutions. Specifically, we turn the smartphone into a beacon device and augment the beacon devices with the capability of forming a mesh network. The implementation result shows that our beacon devices can detect the presence of specific users at specific locations, and then the presence state can be sent to the application server via the relay of beacon devices. Moreover, the application server can send personalized location-based messages to the users, again via the relay of beacon devices. With the capability of relaying messages between the beacon devices, it would be convenient for developers to implement a variety of interactive applications such as tracking VIP customers at the airport, or tracking an elder with Alzheimer’s disease in the neighborhood.

## 1. Introduction

With the advancement of microelectromechanical and wireless communication technologies, the industry of Internet of Things (IoT) is flourishing at the world-wide scale. According to a recent forecast report by Gartner [1], the number of installed IoT devices will be exceeding 25 billion in 2021. Popular application scenarios of IoT include smart homes, smart hospitals, smart factories, and smart cities, to name a few. In these application scenarios, a huge number of IoT devices must be deployed as the infrastructure. To ease the interconnection among IoT devices and the backend systems, a number of low-power wireless communication protocols have been standardized for IoT, such as ZigBee [2], Bluetooth low energy (BLE) [3], Wi-Fi HaLow [4], LoRa [5], etc. Both ZigBee and BLE work in the 2.4 GHz ISM band for short-range communication, while Wi-Fi HaLow and LoRa aim at providing longer reach for IoT devices. Although differences exist, these protocols share a common objective—making the power consumption of wireless communication as low as possible in order to extend the lifetime of battery-operated IoT devices.

Among these protocols, we see the advantage of BLE based on the fact that it is universally supported by almost every smartphone. That means if IoT devices communicate with each other using BLE, people can also communicate with these IoT devices intuitively through their smartphones. By contrast, if IoT devices run ZigBee, smartphones are unable to communicate with them directly because smartphones are without built-in ZigBee wireless interfaces. Solutions to this problem include augmenting the smartphone with a ZigBee USB dongle (e.g., [6]), or using a gateway device to bridge the ZigBee network and the Wi-Fi network (e.g., [7]) or the BLE network (e.g., [8]). Therefore, in this research we focus on naturally integrating BLE-enabled smartphones into the ecosystem of the BLE-based IoT environment by designing an interactive real-time locating system.

BLE was first introduced to the Bluetooth specification in version 4.0 in 2010. It has become the most popular technology for low-power and short-range wireless communication in the application domain of consumer electronics. Compared with the classic Bluetooth (also known as Bluetooth Basic Rate/Enhanced Data Rate, or BR/EDR) which achieves high-speed transmission, BLE technology focuses on greatly reducing the power consumption and provides extremely low transaction latency with only a few milliseconds [9]. BLE-based IoT devices can be powered by coin cell batteries and run for several years on a moderate duty cycle without replacing the battery. Some studies compared the power consumption of BLE and ZigBee and found that BLE is more power-efficient than ZigBee [10,11].

Based on the BLE technology, in 2013 Apple Inc. announced the iBeacon protocol [12] for the purpose of building location-aware applications. Specifically, an iBeacon message is a BLE advertising packet that contains a 16-byte universally unique identifier (UUID) and two 2-byte Major and Minor values. On receiving an iBeacon message, the receiver then uses these values to identify the iBeacon device which broadcasts the iBeacon message. This capability can then be used to implement a variety of location-based services such as determining the current physical location or distributing messages at specific points of interest. Although the iBeacon protocol is useful in providing unidirectional communication from the beacon devices to the smartphones, we find it helpful in some use cases if the reverse communication path exists (i.e., from smartphones to the beacon devices). Currently, to respond to the beacon messages, the smartphone users need to rely on their own mobile Internet connections to communicate with the backend system. Although the mobile Internet services are popular these days, in some cases they may not be always available or too costly. Even if there is a free Wi-Fi service, users are often required to log on before they can use the service. The inconvenience motivates us to design an interactive real-time locating system that only relies on the BLE technology, in which beacon devices not only broadcast beacon messages, but also receive and relay messages from the users. On receiving a message from a specific user, our beacon device detects the presence of the user, and then forwards the presence state to the backend system via the Bluetooth mesh network consisting of beacon devices. By combining the broadcast and mesh topologies in the BLE network, our experimental system can serve as an infrastructure for developers to implement a variety of interactive applications such as tracking VIP customers at the airport, or tracking an elder with Alzheimer’s disease in the neighborhood.

Let us describe a possible use case of our system. An airport company sets up a real-time locating system by deploying a sufficient number of beacon devices in the airport building. The locating service as part of the airport infrastructure can then be leased to airline operators. When a VIP customer of a specific airline arrives at the parking area, he opens the smartphone app provided by the airline. By receiving the beacon advertisements, the app performs locating and then pinpoints the customer on the floor plan. At the same time, the app broadcasts the presence message of the customer which includes the encrypted user ID and his current location. As soon as any beacon device discovers the presence message, it immediately forwards the presence message hop-by-hop over the Bluetooth beacon network to the backend server. As a consequence, the backend server is notified that the VIP customer has arrived at the parking area. The backend server then responds with a customer-specific welcome message and sends it to the smartphone of the VIP customer over the Bluetooth beacon network, letting him know that the airline is aware of his presence and may send a staff to assist him. Through the Bluetooth beacon network, the airline can also provide other information to the VIP customer such as his flight status, or offer him some shopping coupons when he is about to pass through the shop having sales promotion. The VIP customer can also ask for help by pressing a button in the app. Since the real-time location of the customer is tracked, even if he has left the location where he asked for help, the airline staff can still reach him at his real-time location.

Overall, this paper makes the following contributions:

- We combine the broadcast and mesh topology options of Bluetooth to extend the applicability of beacon solutions. Smartphone users are able to interact with the backend system with a single and pervasive network technology—Bluetooth.
- The user experience of Bluetooth beacon-based applications is enhanced because our system can work without smartphone users’ Internet connections. In other words, smartphone users only need to turn on the Bluetooth interface to enjoy the interactive locating services without paying a dime on their phone bills.
- We implemented a real-time locating system solely based on Bluetooth. The feasibility and usefulness of our work have been verified through extensive experiments.
- We identify some potential problems of deploying the Bluetooth locating system, and propose possible solutions to solve/mitigate the problems.

The remainder of this paper is organized as follows. In Section 2 we will discuss related work. In Section 3 we will describe the system architecture and explain the flow of operations. Our implementation details, experimental results, and some implementation issues are described in Section 4. Finally, Section 5 concludes our work.

## 2. Related Work

In this section we review the literature in the field of BLE-based locating systems and applications, as well as some existing implementations of Bluetooth mesh networks. We also give a brief introduction to the Bluetooth beacon technology.

### 2.1. Locating Systems and Applications Based on Bluetooth Low Energy (BLE)

There are a number of related researches in the field of BLE-based locating systems and applications in the literature. Filippoupolitis et al. used BLE beacons to estimate the occupancy of areas inside a building for managing an emergency situation such as fire or terrorist attack [13]. The mobile application installed on the occupant’s mobile device keeps sending the received beacon IDs and their Received Signal Strength Indicator (RSSI) values to a remote control server, by which the server can estimate the location of the occupant. Lin et al. aim at solving the problem of an overcrowded emergency room in which patients waiting to be treated may move around in the hospital. Their solution is a mobile indoor positioning system based on iBeacon to estimate patients’ real-time locations [14], so doctors and nurses can find their patients conveniently. Zhao et al. used iBeacon for warehouse management that improves the performance of the order-picking and stock-taking process with high accuracy [15]. Specifically, the items kept in the warehouse are attached with beacon transmitters, and the beacon receivers are deployed in the warehouse. When searching for a specific item, the warehouse management system can locate the item by RSSI fingerprint matching. Faragher and Harle [16] explored fingerprint positioning using BLE beacons. Their experimental result showed that with relatively sparse deployment of beacons, using BLE has better positioning accuracy compared with using Wi-Fi. Onofre et al. also studied the topic of indoor positioning using BLE beacons [17]. Specifically, they tried to minimize the error between real distance and estimation by using fuzzy logic. At several different distances, the average positioning errors of using fuzzy logic are lower than using direct RSSI readings and median values. Fard et al. proposed a light-weight indoor positioning algorithm based on RSSI fingerprint matching [18]. To accelerate the process of fingerprint matching, they used a reduced-degree matrix composed of only 3-tuples (average, count, and standard deviation of the non-zero signal readings), and the experimental results showed good positioning accuracy. Wu et al. improved the accuracy of inertial sensor-based indoor localization with the help of iBeacon devices [19]. In their design, when a user enters the vicinity of iBeacon device clusters, the accumulated errors of the inertial sensor-based indoor localization can be corrected by iBeacon localization. Experimental results show that 90% of the localization errors are within 3.5 m in large open areas. Martin et al. focused on investigating the accuracy of distance estimation using iBeacon [20]. They also designed a mobile user interface (UI) for users to adjust the beacon settings (TX power and frequency) in order to witness how the settings can affect the distance estimates. To further improve the accuracy of indoor localization, He et al. proposed a novel technique called *INTRI*, which employs trilateration in fingerprint-based environment [21]. First, in the environment the signal strengths from each access point are measured in order to form distinct contours of the equal signal strengths in the fingerprint region. Then, given the user’s received signal vector, the user’s location can be correlated with a number of contours, and accurately located by the concept of trilateration. Their proposed technique is a general approach applicable to various fingerprint signals such as Wi-Fi, ZigBee, RFID, and Bluetooth.

### 2.2. Implementations of Bluetooth Mesh Networking

In Section 1 we have mentioned that the beacon technology only provides unidirectional communication. Although Bluetooth supports bidirectional communication, at the time of our implementation the communication is limited to a star topology. In July 2017 the Bluetooth Special Interest Group (SIG) finally released the official Bluetooth mesh networking specifications [22]. Before the Bluetooth mesh networking specifications were released, chip vendors and researchers have devoted themselves to developing mesh protocols for BLE. In 2014 a proprietary flood-based mesh protocol for BLE called CSRmesh was released by CSR Inc. (acquired by Qualcomm in 2015) [23]. CSRmesh supports up to 65535 nodes per network, and the propagation delay between nodes is less than 15ms. Zenker et al. studied the packet delivery ratio of different setups using CSRmesh protocol [24]. They found that although using the flood-based approach has the advantage of simplicity over route-based approaches, flooding does not work well in traffic-intensive application scenarios. nRF OpenMesh [25] is also a flood-based mesh protocol for BLE, which is developed by Nordic Semiconductor Inc. In nRF OpenMesh, the Trickle algorithm [26] is used to dynamically control the rebroadcasting behavior based on node density and value update frequency. An opportunistic routing (OR)-based mesh protocol called BLEmesh [27] was proposed by Kim et al. In BLEmesh the source node broadcasts the packets and selects the next forwarding nodes among those who successfully received the packets in an opportunistic manner. Compared with conventional routing protocols for wireless mesh networks, using OR requires a smaller number of broadcast packets to finish the transmission, which also means less power is consumed.

### 2.3. Bluetooth Beacon Technology

The Bluetooth beacon technology has gained significant attention in providing proximity-aware services for consumers, businesses, and industrial environments. There are mainly three “pseudo-standards” for Bluetooth beacons: iBeacon [12], Eddystone [28], and AltBeacon [29], proposed by Apple, Google, and Radius Networks, respectively. All three pseudo-standards are based on broadcasting BLE advertising packets, which carry specific information in the payload area. When a Bluetooth scanner receives an advertising packet, it decodes the content and takes corresponding actions. The message format of iBeacon consist of 4 fields: UUID, Major, Minor, and TX power. By comparing the received signal strength of the iBeacon message and the value of TX power in the message, the receiver can determine the approximate proximity to the iBeacon transmitter. For Eddystone, it has four message formats served for different purposes: Eddystone-UID, Eddystone-EID, Eddystone-TLM, and Eddystone-URL. Take Eddystone-URL as an example, this type of message is used to broadcast an URL that redirects the receiver to a website, which realizes the so-called physical web [30]. AltBeacon was designed for an open and interoperable specification. The message format of AltBeacon consists of Manufacturer ID, Beacon Code, Beacon ID, and Reference RSSI. Compared with iBeacon, an AltBeacon message has more user data bytes, which delivers more data per message.

## 3. System Design

Since the release of Bluetooth mesh networking specifications in July 2017, Bluetooth has three topology options: point-to-point, broadcast, and mesh [31]. Point-to-point connections are commonly used for audio streaming or data exchange between a smartphone and a headset or a smart watch. Broadcast topologies offer one-to-many device communications, which is well-suited for beacon solutions. Mesh topologies allow many-to-many device communications, which is ideal for building sensor network applications. In this work, we combine the latter two topology options—broadcast and mesh—to extend the applicability of beacon solutions. Specifically, the unique feature of our system is that we augment the BLE beacon devices with the capability of forming a mesh network, so interactive applications inside the beacon mesh network can be fulfilled solely based on the transport of the BLE technology. In the following, we will describe the system architecture, the principle of operation, and the design of messages in the beacon network.

### 3.1. System Architecture

**Figure 1:**
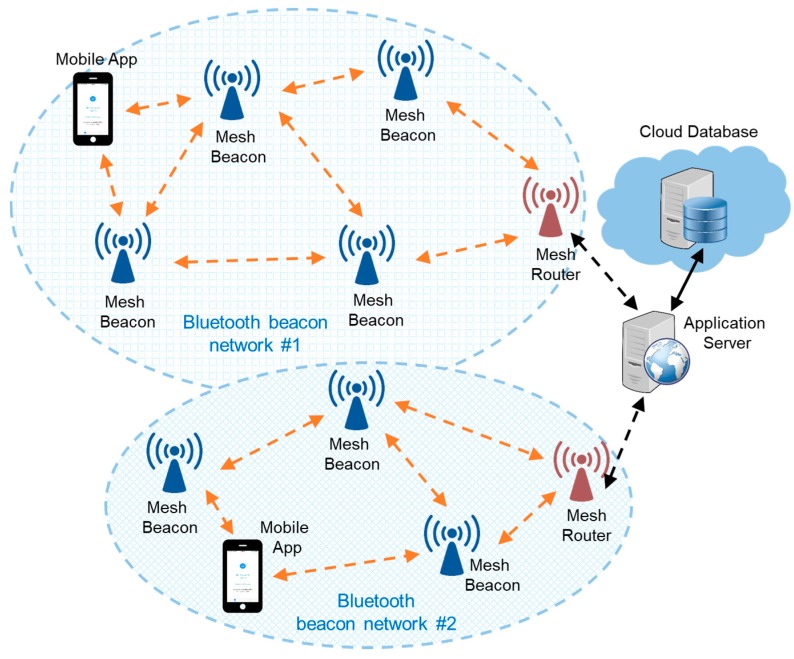
System architecture.

As shown in Figure 1, the overall system consists of five roles: Mobile App, Mesh Beacon, Mesh Router, Application Server, and Cloud Database. A number of Mesh Beacons and a Mesh Router form a Bluetooth beacon network. The five roles are elaborated as follows:

1. Mobile App: a locating application running on mobile users’ smartphones. It listens to the beacon messages sent by the Mesh Beacons to figure out the current location of the mobile user. It responds to the received beacon message by broadcasting the mobile user’s encrypted presence message.
2. Mesh Beacon: a beacon device that can send, receive, and relay messages in the Bluetooth beacon network. Specifically, it not only broadcasts beacon messages, but also receives presence message from Mobile Apps, and relays the presence message to the Application Server.
3. Mesh Router: a gateway device between the Bluetooth beacon network and the rest of the network. It is responsible for collecting the presence messages from the Mobile Apps within the range of the Bluetooth beacon network. Once the Mesh Router receives a presence message, it will upload the presence state to the Application Server through hypertext transfer protocol (HTTP), and send an acknowledgement message back to the Mobile App in response to receiving the presence message from a specific user.
4. Application Server: a shared HTTP server for the Mesh Routers from several Bluetooth beacon networks. It buffers the presence states of the mobile users, and forwards the presence states to the Cloud Database.
5. Cloud Database: a real-time database in the cloud environment. It is responsible for keeping the history records of all the mobile users’ presence states.

In Figure 1 we can see two Bluetooth beacon networks, in which all communications take place over the transport of the BLE technology. As for the communications outside the Bluetooth beacon networks, one may choose whatever technologies are available such as ethernet or Wi-Fi. In our system, the Mesh Router connects to the Application Server by using its built-in Wi-Fi interface.

### 3.2. Flow of Operations

In this subsection we describe the flow of operations of our system. When the Mesh Beacons are powered on, they periodically broadcast beacon messages over the advertising channel, just like ordinary beacon devices do. Moreover, our Mesh Beacons also listen to the messages carrying users’ presence state from the Mobile Apps at the same time. In the following, we illustrate the flow of operations when a smartphone user with our Mobile App enters the communication range of our Bluetooth beacon network.

1. When the Mobile App discovers a beacon message from a certain Mesh Beacon, it locates itself based on the Major/Minor values embedded in the message. In our design, the Mobile App directly looks up a built-in location table to resolve the Major/Minor values, so the name of the detected location can be shown on the user interface immediately.
2. Once the locating process has completed, the Mobile App composes the presence message containing the encrypted user ID and the detected Major/Minor values, and then broadcasts the presence message. The objective of broadcasting the presence message is to inform the backend system that this specific user has shown up at a particular location.
3. As soon as any Mesh Beacon hears the presence message, it uses the managed-flood-based approach to rebroadcast the presence message. The purpose of rebroadcasting the presence message is to relay it to the Mesh Router. The managed-flood-based approach can make sure that once a specific presence message has been broadcasted by a Mesh Beacon, the Mesh Beacon will not rebroadcast it again.
4. When the Mesh Router hears the presence message originated from a certain Mobile App, it uploads the content in the presence message to the Application Server. Meanwhile, it sends an acknowledgement message back to the Mobile App by broadcast.
5. When any Mesh Beacon hears the acknowledgement message, it rebroadcasts the message by using the managed-flood-based approach again. The purpose of rebroadcasting the acknowledgement message is to relay it to the Mobile App.
6. (Continue from item 4) When the Application Server receives the presence state from the Mesh Router, it forwards the presence state to the Cloud Database. In the Cloud Database we are able to see the locating history for all the mobile users.
7. When the acknowledgement message from the Mesh Router has been received at the Mobile App, the Mobile App displays a welcome message indicating that the backend system is aware of the user’s presence.

**Figure 2:**
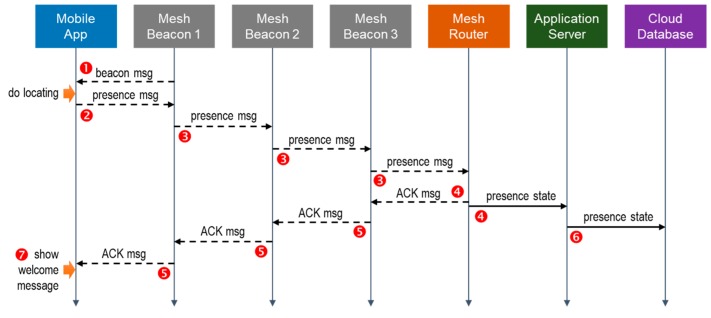
An example of the flow of operations.

The sequence diagram in Figure 2 shows an example of the flow of operations, where the labeled numbers correspond to the 7 items described above. In this example, there are three Mesh Beacons on the transmission path between the Mobile App and the Mesh Router. From the diagram, we can see how the interactivity between the mobile user and the backend system is achieved. From the users’ perspective, they are able to receive more than the basic locating service. Benefiting from interacting with the backend system over a single Bluetooth beacon network, they can enjoy personalized services without having to connect to the Internet. From the system operators’ perspective, they are able to detect the presence of specific users at particular locations, and then offer personalized services to them. With the presence state stored in the Cloud Database, the system operators can also track and analyze user behaviors using the users’ real-time and past locations.

### 3.3. Messages in the Beacon Network

**Figure 3:**
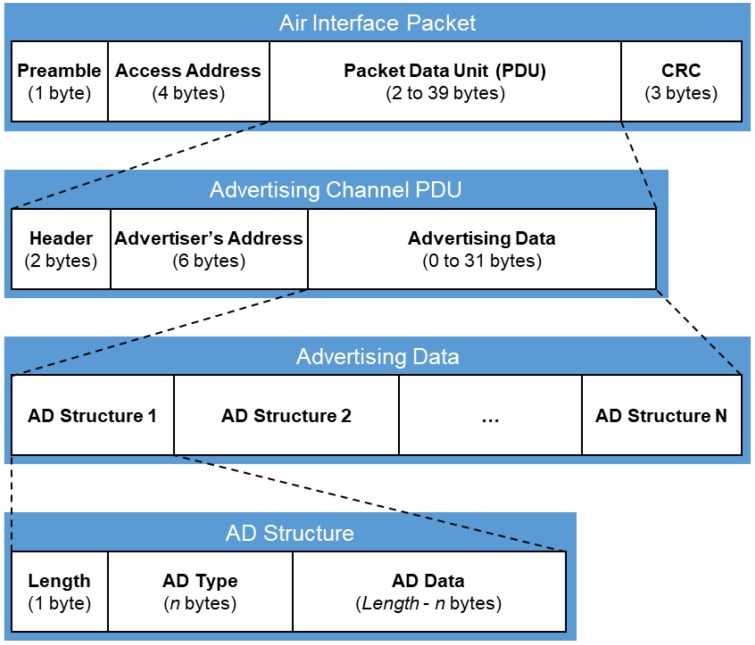
The breakdown of a Bluetooth low energy (BLE) air interface packet.

**Figure 4:**
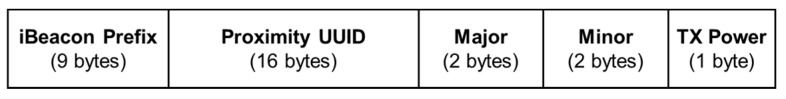
Apple iBeacon message format.

In our system, we use BLE non-connectable advertising packets to implement all types of messages so there would be no need for the devices to pair with each other before sending any data. The breakdown of a BLE air interface packet is shown in Figure 3. According to Bluetooth Specification v4.1 [32], the maximum size of a BLE advertising packet data unit (PDU) is 39 bytes. Excluding the 2-byte header field and the 6-byte advertiser’s address field, there are up to 31 bytes to carry the advertising data. The advertising data is further divided into a sequence of advertising data (AD) structures. The iBeacon protocol uses AD Structure 1 and AD Structure 2 combined to carry its advertisement information, with the message format shown in Figure 4.

There are four types of messages in our Bluetooth beacon network:

- Type 1: beacon messages sent by the Mesh Beacons
- Type 2: presence messages sent by the Mobile Apps
- Type 3: presence messages forwarded by the Mesh Beacons
- Type 4: acknowledgement messages sent by the Mesh Router.

**Figure 5:**
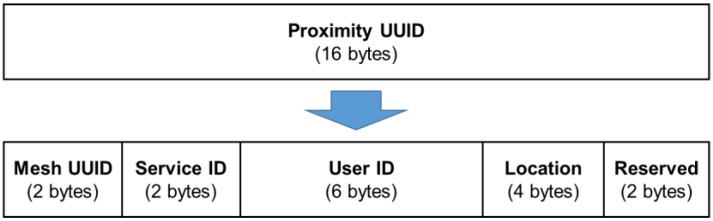
User’s presence state from the Mobile App is embedded in the Proximity universally unique identifier (UUID) field of a standard iBeacon message.

First, in our implementation the beacon message (Type 1) format follows the iBeacon format, where the Proximity UUID field is filled with our own 128-bit Mesh UUID. We configure the Mesh Beacons to broadcast beacon messages every 100 ms. Next, for the design of the presence message sent by the Mobile App (Type 2), we are forced to follow the iBeacon message format again because we implement our Mobile App on the iOS platform. Since we are not allowed to customize the message format, our solution is to embed the presence state in the Proximity UUID field. Specifically, we reformat the 16-byte Proximity UUID field into 5 fields shown in Figure 5. The Mesh UUID value is set to 0x7ECE, which is the 16-bit prefix of our 128-bit Mesh UUID. The Service ID is designed to indicate the message type. For the presence message from the Mobile App (Type 2), the Service ID is assigned the value of 0xD122. The User ID field carries the encrypted user ID, which is determined at the time the user registered. The Location field carries the discovered Major/Minor values from the received beacon message. Note that in Type 2 messages, the values in the original Major/Minor fields of the iBeacon message are unused since Mesh Beacons need not to do locating on receiving Type 2 messages.

**Figure 6:**
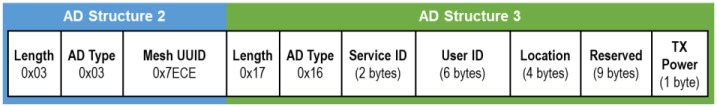
The message format for Type 3 and Type 4 messages.

As for the presence messages forwarded by the Mesh Beacons (Type 3) and the acknowledgement messages sent by the Mesh Router (Type 4), we are able to customize their formats using the BLE advertising packet. Basically, they share the same format as shown in Figure 3 with three AD structures. To simplify the presentation, in Figure 6 we only show AD Structure 2 and 3 in our design. In AD Structure 2, the AD Type of 0x03 means that there is a 16-bit Service Class UUID in the AD Data field. We use this field to denote the Mesh UUID, so the value in this field is also 0x7ECE. In AD Structure 3, we see the Service ID field again. To distinguish the two types of messages from the presence message sent by the Mobile App (Type 2), the presence message forwarded by the Mesh Beacon (Type 3) is assigned the value of 0xD123, and the acknowledgement message sent by the Mesh Router (Type 4) is assigned 0xD124, respectively. Finally, the User ID and the Location fields serve the same functionalities as in Figure 5, which have been described in the previous paragraph.

As we stated earlier, the Mesh Beacons are configured to broadcast beacon messages (i.e., Type 1 messages) periodically. When the Mobile App discovers a beacon message, it first checks whether the carried Mesh UUID matches our own specific value. If it is a match, the Mobile App uses the discovered Major/Minor values to locate itself immediately. Once the locating process is successful, the Mobile App will send the mobile user’s presence state by broadcasting a presence message (i.e., a Type 2 message). In the presence message, the Mobile App specifies the encrypted user ID and the Location (i.e., the received Major/Minor values), meaning that the user is nearby the location associated with the Major/Minor values. Once a Type 2 message is detected by any Mesh Beacon, the Mesh Beacon uses the embedded User ID and Location values to construct a Type 3 message, and then broadcasts the Type 3 message. For all other Mesh Beacons that discover the Type 3 message, they use the managed-flood-based approach to relay it. When the Type 3 message finally reaches the Mesh Router, the Mesh Router sends the encrypted user ID and the Major/Minor values to the Application Server over HTTP. At the same time, the Mesh Router uses the received Type 3 message to compose a Type 4 message by simply changing the Service ID to 0xD124, and then broadcasts the message. With the help of Mesh Beacons, the Type 4 message is forwarded over the Bluetooth beacon network to the Mobile App. On receiving the Type 4 message with its own user ID, the Mobile App will show a message box indicating that the application server has been notified of the presence of the user. At the Application Server, the Major/Minor values are translated into the name of the associated location, and then the user’s presence state is sent to the Cloud Database. Eventually, with the presence state of all the mobile users in the database, we are able to analyze the locating history and to create our own innovative applications.

## 4. Implementation and Experiments

In this section, we will first show the implementation details of our system, including the hardware and the software. Then, we will describe the experiments to verify the operations of our system. Finally, we will discuss some implementation issues when deploying our system.

### 4.1. Implementation and Deployment of Our Prototype System

**Figure 7:**
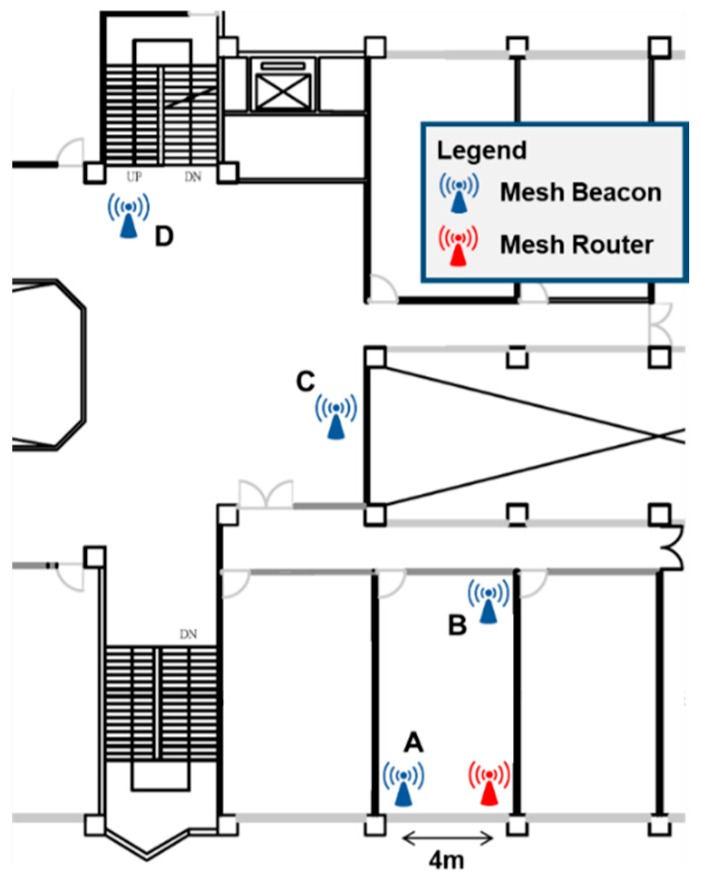
Deployment of BLE beacon network with 4 Mesh Beacons and 1 Mesh Router.

Table 1 lists the hardware and software used in our prototype system. Our Mobile App is developed to run on Apple iOS. Mesh Beacons and Mesh Routers are implemented using the RedBear development board [33] because of its small size and the co-existence of BLE and Wi-Fi interfaces. The Application Server is an Ubuntu PC with a Node.js web server. The Cloud Database is implemented using Google Firebase [34], which also provides the user registration and authentication functionalities for our Mobile App. Our BLE beacon network is deployed on the 8th floor of the engineering building in our campus. Figure 7 shows the floor plan where we deployed four Mesh Beacons (the blue ones labeled *A* through *D*) and one Mesh Router (the red one).

**Figure 8:**
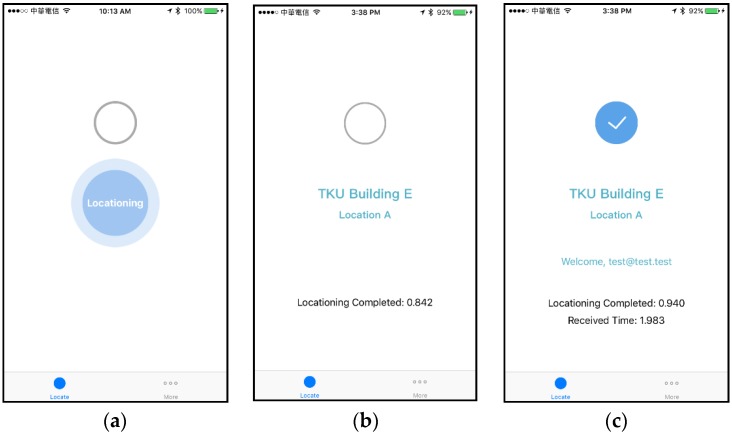
Screenshots of our Mobile App (**a**) during the locating process (**b**) physical location determined (**c**) acknowledgement message received.

First, we describe the user interface of the Mobile App because we use this to measure and display the response time. Three screenshots of our Mobile App are shown in Figure 8. After the user logs in and then manually presses the “Locate” button, the central “Locationing” circle with breathing light effect will appear as in Figure 8a. During this locating period, the Mobile App scans for the beacon messages sent by Mesh Beacons. As soon as a beacon message is received and the physical location is determined, the location name will show up as in Figure 8b, which is “TKU Building E Location A” in this case. Meanwhile, the Mobile App will broadcast the user’s presence state into the Bluetooth beacon network. In response to the presence message from the Mobile App, the Mesh Router will send an acknowledgement message back to the Mobile App. When the acknowledgement message finally arrives at the Mobile App, a welcome message shows up and the check mark appears as in Figure 8c, meaning the whole process has now completed. Regarding the response time, the time (0.842 s) shown in Figure 8b refers to the period starting from the user pressed the “Locate” button, to the time when the physical location is determined at the Mobile App. Note that the screenshot in Figure 8c was captured in another test. The received time (1.983 s) shown in Figure 8c refers to the period starting from the completion of locating, to the instant that the Mobile App received the acknowledgement message from the Mesh Router.

### 4.2. Experiment 1

**Figure 9:**
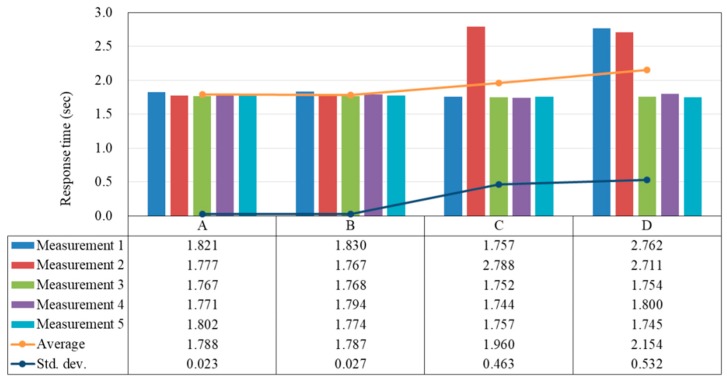
Time taken for the Mobile App to receive the acknowledgement message in Experiment 1 (s).

In this experiment, we would like to measure the response time from the Mobile App users’ perspective. Specifically, we put the smartphone beside the Mesh Beacons and then open the Mobile App. At each of the four locations (*A* through *D*), we measure the response time five times, and show the results in Figure 9. Note that since the locating process is based on an internal table lookup in the Mobile App, the time needed to finish the locating process does not depend on the real locations. Therefore, in Figure 9 we only show the time taken for the Mobile App to receive the acknowledgement message.

From Figure 9 we can see that at both locations *A* and *B* the observed response times are relatively stable, but at locations *C* and *D* some of the response times are more than 2.7 s. The reason is that at locations *A* and *B*, they are only one hop away from the Mesh Router; while at locations *C* and *D*, they are 2 and 3 hops away, respectively. It is straightforward that with a greater number of hops on the path, the probability of a lost presence message or acknowledgement message is also higher. Furthermore, we need to describe the behavior of the Mobile App in order to justify the result. In our implementation, the Mobile App takes about 0.7 s to initialize the advertisement process, and then proceed to advertise the presence message for 1 s. After that, the Mobile App will start listening to the acknowledgement message from the Mesh Router. This explains why the observed minimum response time is around 1.7 s. If no acknowledgement message is received within 1 s, the Mobile App will advertise the presence message again for another 1 s. Knowing the behavior of the Mobile App, we can conclude that at locations *A* and *B*, the Mobile App successfully received the acknowledgement message after the first advertisement period. However, from the measurement results at locations *C* and *D*, we can find that some of the acknowledgement messages arrived after the second advertising period. Nevertheless, we think that the response time can be well-controlled if we place any two neighboring Mesh Beacons within a reasonable distance to keep the packet loss rate low. Furthermore, it is also possible to reduce the response time by shortening the advertising period and the waiting period because this is simply an implementation issue. Since the 0.7-s initialization process is inevitable, we expect that in the best case the minimum response time can be within 1 s.

### 4.3. Experiment 2

**Figure 10:**
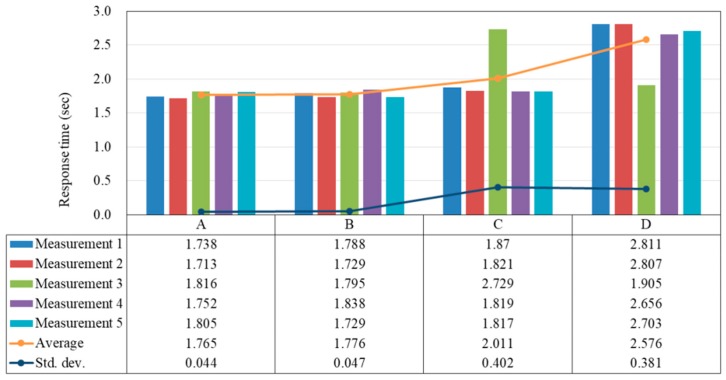
Time taken for the Mobile App to receive the acknowledgement message in Experiment 2 (s).

Experiment 2 basically follows the design of Experiment 1. However, the difference is that we focus more on the multi-hop relay function of the Mesh Beacons and measure the response time. Specifically, in this experiment, if we stand at location *D*, then only Mesh Beacon *D* is configured to receive the presence message from the Mobile App (i.e., Type 2 message), while Mesh Beacons *A*, *B*, and *C* are forced to ignore Type 2 messages. This ensures that when the user is physically located at location *D*, its presence message is only received by Mesh Beacon *D* rather than by the nearby Mesh Beacon *C*, which has less number of hops to the Mesh Router. The same idea applies to all other cases, in which only the Mesh Beacon closest to the user is able to receive the presence message from the Mobile App. Again, at each of the four locations, we measure the response time for five times and show the result in Figure 10.

As expected, the above experimental results are almost identical with that of Experiment 1, with the exception of the measurement results at location *D*. Specifically, here the majority of the response times at location *D* are around 2.7 s, while in Experiment 1 only two measurements exceed 2.7 s. This reveals the fact that although the user is physically closer to Mesh Beacon *D*, in Experiment 1 some of the presence message from the Mobile App must have been received and then forwarded by the nearby Mesh Beacon *C* so a better performance (fewer packet losses) can be observed. Moreover, if we compare the measurement results at location *C* of the two experiments, we can find that the average response time in Experiment 2 is slightly higher than that in Experiment 1. We speculate that this is because in Experiment 1, when the user is standing beside Mesh Beacon *C*, some of the presence messages may have been received and then forwarded by either Mesh Beacon *A* or Mesh Beacon *B*. Therefore, some of the measurement results are on the 2-hop distance (round-trip). However, in Experiment 2 the round-trip distance from Mesh Beacon *C* to the Mesh Router is 4 hops in every measurement. This is the reason why the average response time becomes higher than that in Experiment 1.

### 4.4. Experiment 3

**Figure 11:**
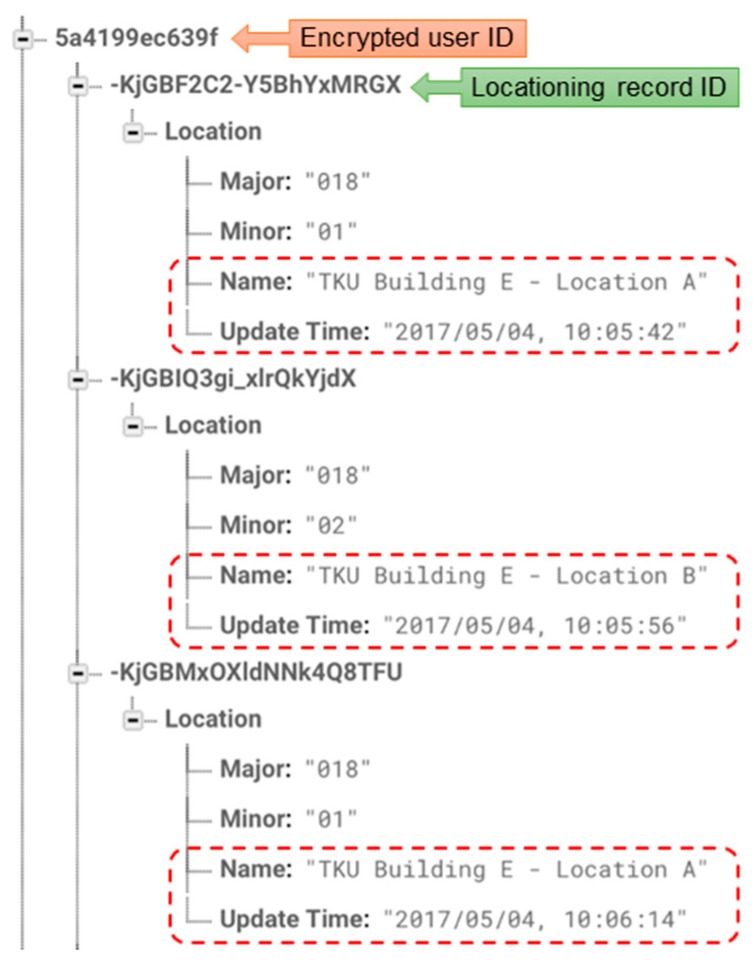
A snapshot of the Firebase database showing the moving pattern of a user.

In this experiment, we check the overall operations of the prototype system. First, we let a user with the Mobile App walk from location *A* to location *B*, and then return to location *A*. As expected, the smartphone user was able to see the correct locating results in the Mobile App which switched from location *A* to location *B*, and then switched back to location *A*. Then we checked the moving pattern recorded in the Cloud Database. Figure 11 is a screenshot from the Google Firebase, in which the top-level key refers to the encrypted ID of the user and the second-level keys refer to the locating records for this user. In each record there are values of Major and Minor, the associated location name, and the timestamp of the locating record. From Figure 11 we can see three consecutive records with the determined location *A*, *B*, and then *A*. The order of the determined locations in the Cloud Database apparently matches the route taken by the user, and the timestamps correctly indicate the time that the user was physically located at those locations.

Next, we test the use case of two simultaneous users. Specifically, to ease the cross check of the locating results, in this test two smartphones were held by a single person to emulate two users taking the same route simultaneously. The route starts from location *B* to location *D* via location *C*, then takes the reverse direction back to location *B*. Table 2 summarizes the observed locating results recorded in the Cloud Database, from which we can compare the timestamps of the two emulated users at these locations. Since the test was carried out by a single person, ideally the locating results of the two emulated users should be exactly the same. However, we can see that there exist time differences in most of the locating results. Again, we believe this is due to the natural uncertainty of multi-hop wireless transmissions and possibly the contention of wireless resource. Fortunately the time differences are limited to only a few seconds and would not be a problem.

### 4.5. Discussions

In this subsection, we discuss some implementation-related issues of the system, including the communication range of Bluetooth beacons, the contention on the BLE advertising channels, the interference from Wi-Fi devices, and how to deal with a large deployment area.

#### 4.5.1. The Communication Range of Bluetooth Beacons

Theoretically the transmission range of Bluetooth 4 can be up to 100 m [35]. In our experimental deployment the maximum distance between neighboring Mesh Beacons is about 8 to 10 m. The reason is that after several field tests, we found that if the distance is greater than 10 m, packet losses would become significant. This is due to the limited transmission range of the development boards we used, as well as the non-line-of-sight deployment of the devices. Since we need to deploy the Mesh Router in our own lab for a stable network connection to the Application Server (a PC in the same lab), we have no choice but to deploy part of the system non-line-of-sight. In real deployments such as airports or exhibition centers, one can choose Bluetooth devices with stronger output power or external antennas to increase the radio coverage, along with proper line-of-sight placement of the Bluetooth devices to improve the transmission quality. Doing so can also reduce the total number of devices needed to cover the whole field of deployment.

#### 4.5.2. Contention on Advertising Channels and Interference from Wi-Fi

We know that Bluetooth beacons are broadcast on the three advertising channels. Since the message relays in the Bluetooth mesh networks are also based on broadcasting, when the number of Mesh Beacons is large, the contention of using the advertising channels may become an important issue. A good solution to this problem is the latest Bluetooth 5.0 [36]. In Bluetooth 5.0, a new physical layer named LE 2M is introduced (note that the physical layer used in Bluetooth 4 is called LE 1M). With LE 2M, its theoretical data rate of 2 Mbit/s can significantly reduce the air time needed to transmit a given amount of data, leading to better spectral efficiency. Moreover, in Bluetooth 5.0 the payload of the advertising packets can be offloaded to the available data channels, leaving only the header data transmitted on the advertising channels. On the problem of possible interference from Wi-Fi communications, Bluetooth 5.0 features an improved channel sequencing algorithm that improves the pseudo randomness of the next hop channel sequencing. This algorithm also improves the co-existence performance of Bluetooth devices in the presence of Wi-Fi devices. When the commercial Bluetooth 5.0 chips are widely available and have been used in beacon systems, we believe that the locating performance of such systems can be greatly enhanced.

#### 4.5.3. Problems Related to a Large Deployment Area

**Figure 12:**
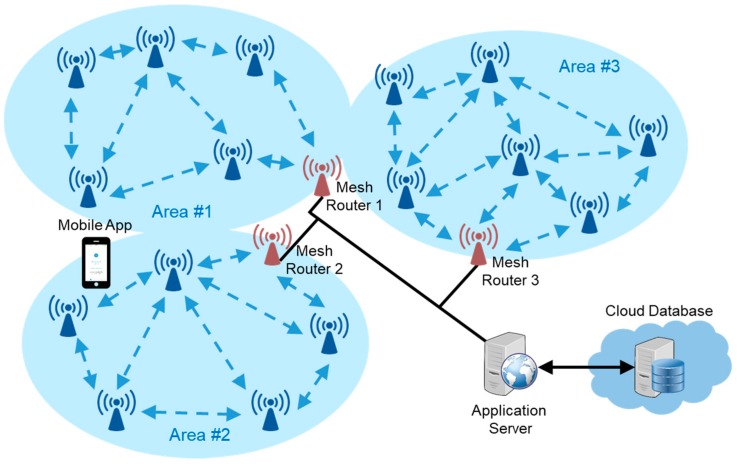
Deployment with multiple subareas to reduce the hop count to the Mesh Router.

When the field of deployment is large, some of the Mesh Beacons will be many hops away from the Mesh Router. When the users are located around those remote Mesh Beacons, they may experience a longer response time owing to greater physical distance and higher packet loss rate. A practicable solution to this problem is to divide the large deployment area into several smaller subareas, and equip each subarea with its own Mesh Router. Mesh Routers, on the other hand, can connect to the Application Server through Wi-Fi or ethernet interfaces. The idea is depicted in Figure 12. Therefore, the number of hops from any Mesh Beacons to its closest Mesh Router can be well-controlled. Besides, Mesh Beacons can be configured not to rebroadcast the messages from different subareas they belong to, so as to reduce the total number of broadcast messages in the deployment field.

Last but not least, when the deployment area is large, it takes more Mesh Beacons to cover the area. With a greater number of Mesh Beacons in the network, the overhead incurred by the broadcast behavior of message relays is also increased. Specifically, although broadcasting avoids the need to create and maintain routes, it may be less efficient from the perspective of the total number of consumed messages to fulfill an end-to-end communication between two devices [37]. From our point of view, the overhead of the broadcast can be controlled if the Bluetooth beacon networks are well-designed. Just like what we have mentioned in the previous paragraph, if the rebroadcasts can be limited inside smaller subareas, the scalability of the Bluetooth beacon networks will not be an issue. In addition, the official Bluetooth mesh protocol also utilizes the managed-flood approach to implement the mesh network. This approach is based on message relays to rebroadcast messages, which is exactly what our Mesh Beacons do. As a matter of fact, our Mesh Beacons do more than acting as message relays—they play the role of reference points for micro-location services at the same time.

## 5. Conclusions

Since the invention of iBeacon and given the extreme popularity of smartphones, the BLE-based beacon technology has been deployed extensively as a basis to provide various locating services for smartphone users. In this research, our main contribution is that we combined the Bluetooth broadcast and mesh topologies to extend the applicability of beacon solutions. Specifically, apart from broadcasting beacon messages, our beacon devices also serve as beacon readers that can discover the presence of specific users, and then forward the presence state hop-by-hop to the backend server over the Bluetooth beacon network. With the knowledge of a specific customer’s presence, the backend server can respond to the customer with a personalized message, again via the relay of the Bluetooth beacon network. In some use cases, such as welcoming a VIP customer at the airport, our interactive locating system can give the customer a much improved user experience, since all the communications rely on a single network technology—BLE. Neither costly mobile Internet connections nor the troublesome Wi-Fi connections are needed for the customers.

Our current implementation of messaging over the Bluetooth beacon network is based on rebroadcasting in the advertising channels. It is easy to implement, and it precludes the need to pair Bluetooth devices before sending messages to each other. Furthermore, without having to run a routing protocol and maintain a routing table, the complexity and memory consumption of the Bluetooth devices can be minimized. Although sending messages by broadcast is less efficient, the overhead can be well-controlled if we design the rebroadcast mechanism carefully as well as dividing a large network into smaller subareas properly. In the future, we plan to replace the Bluetooth interfaces with version 5.0 and incorporate the official Bluetooth mesh protocol into our system, to achieve better locating performance and the interoperability between BLE-based mesh networks.

## Figures and Tables

**Table 1:** Hardware and software used in our prototype system.

Item	Technology
Mobile App	Apple iOS version 10.2
Mesh Beacon and Mesh Router	RedBear Duo development board (with Bluetooth V4.1 and IEEE 802.11n Wi-Fi)
Application Server	Node.js web server on Linux Ubuntu 14.04
Cloud Database	Google Firebase

**Table 2:** Locating results of two simultaneous users recorded in the Cloud Database.

Locations	Timestamp of the Locating Record	
	User 1	User 2
*B*	17:14:18	17:14:18
*C*	17:14:37	17:14:36
*D*	17:14:56	17:14:58
*C*	17:15:24	17:15:27
*B*	17:15:39	17:15:40
